# A feasibility randomized controlled trial of a NICU rehabilitation program for very low birth weight infants

**DOI:** 10.1038/s41598-022-05849-w

**Published:** 2022-02-02

**Authors:** Lisa Letzkus, Mark Conaway, Claiborne Miller-Davis, Jodi Darring, Jessica Keim-Malpass, Santina Zanelli

**Affiliations:** 1 Division of Developmental Pediatrics, Department of Pediatrics, University of Virginia, Charlottesville, VA USA; 2 Public Health Sciences, University of Virginia, Charlottesville, VA USA; 3 University of Virginia Medical Center, Charlottesville, VA USA; 4 School of Nursing, University of Virginia, Charlottesville, VA USA; 5 Division of Neonatology, Department of Pediatrics, University of Virginia, Charlottesville, VA USA; 6 University of Virginia Children’s Hospital, PO BOX 800828, Charlottesville, VA 22908 USA

**Keywords:** Neonatal brain damage, Neonatology, Paediatric research, Preterm birth

## Abstract

Motor disability is common in children born preterm. Interventions focusing on environmental enrichment and emotional connection can positively impact outcomes. The NICU-based rehabilitation (NeoRehab) program consists of evidence-based interventions provided by a parent in addition to usual care. The program combines positive sensory experiences (vocal soothing, scent exchange, comforting touch, skin-to-skin care) as well as motor training (massage and physical therapy) in a gestational age (GA) appropriate fashion. To investigate the acceptability, feasibility and fidelity of the NeoRehab program in very low birthweight (VLBW) infants. All interventions were provided by parents in addition to usual care. Infants (≤ 32 weeks' GA and/or ≤ 1500 g birthweight) were enrolled in a randomized controlled trial comparing NeoRehab to usual care (03/2019–10/2020). The a priori dosing goal was for interventions to be performed 5 days/week. The primary outcomes were the acceptability, feasibility and fidelity of the NeoRehab program. 36 participants were randomized to the intervention group and 34 allocated to usual care. The recruitment rate was 71% and retention rate 98%. None of the interventions met the 5 days per week pre-established goal. 97% of participants documented performing a combination of interventions at least 3 times per week. The NeoRehab program was well received and acceptable to parents of VLBW infants. Programs that place a high demand on parents (5 days per week) are not feasible and goals of intervention at least 3 times per week appear to be feasible in the context of the United States. Parent-provided motor interventions were most challenging to parents and alternative strategies should be considered in future studies. Further studies are needed to evaluate the relationship between intervention dosing on long term motor outcomes.

## Introduction

Prematurity is a major problem in the United States where 1 in 10 newborns are born preterm and 1 to 2 in 100 are born very preterm (less than 32 weeks’ gestation)^1^. While the survival rates continue to improve, even in the most immature infants, neurodevelopmental impairments have not improved over several decades^2–5^. Cerebral Palsy (CP) is the most common motor disability affecting 7–20% of children born preterm^6^. In 2017, international early detection of CP guidelines were published to provide recommendations on available tools and imaging to best identify patients at risk for CP^7^. These guidelines highlight the role of standardized assessment tools such as the Hammersmith Infant Neurological Evaluation (HINE) and the General Movement Assessment (GMA)^7^.

The frequency and severity of neurodevelopmental impairments is inversely correlated with gestational age (GA). GA also informs the risk of major NICU morbidities including brain injury, a major predictor of abnormal neurodevelopment. Further, increasing evidence supports the adverse impacts of preterm birth and the NICU environment on the trajectory of brain development, even in the absence of overt brain injury. Multiple studies demonstrate changes in brain volume, white matter development, cortical folding and measures of connectivity in infants born preterm^8–10^. Recent evidence also suggest that preterm birth may impact cognitive reserve and brain aging^11,12^. The NICU can be characterized as a toxic environment with excess exposure to inflammation, hypoxia–ischemia, pain, stress, noise, light, as well as deprivation in meaningful social interaction and language, comforting touch and sleep. As such, strategies designed to mitigate this impact and promote the optimal motor and cognitive development of high-risk preterm infants are a high research priority.

Multiple NICU-based developmental programs that focus on positive sensory interventions to improve outcomes have been investigated, including the Auditory-Tactile-Visual-Vestibular (ATVV) intervention, Creating Opportunities for Parent Empowerment (COPE), Family Nurture Intervention (FNI) strategy, Newborn Individualized Developmental Care and Assessment Program (NIDCAP) and Supporting and Enhancing NICU Sensory Experience (SENSE) programs^13–17^. While these programs indicate that early interventions can improve short-term outcomes, there remains gaps in knowledge regarding the optimal timing, dosing and type of interventions required to prevent the risk of motor impairment such as CP. And as such, there is no agreed upon best practice for early, NICU-based interventions for high risk preterm infants. Therefore, a logical first step is to evaluate the feasibility of multisensory, parent-provided NICU-based interventions.

The objective of this study was to investigate the acceptability, feasibility and fidelity of a NICU-based rehabilitation program designed to provide positive sensory experiences (vocal soothing, scent exchange, comforting touch, skin-to-skin care) as well as motor training (massage and physical therapy) for very low birthweight (VLBW) infants. All interventions were provided by parents in a GA appropriate fashion and in addition to usual care. A secondary aim was to describe usual care for those randomized to the control group.

## Methods

This randomized controlled trial (RCT) was conducted at the University of Virginia (UVA), a level IV academic NICU. The study was registered at clinical trials.gov (clinical trials.gov identifier: NCT04330859, first submitted 29/04/2019, registered retrospectively 02/04/2020). All methods were performed in accordance with relevant guidelines and the study was approved by the University of Virginia Health Sciences Research Institutional Review Board approval prior to starting any study procedures. Informed written consent from a legal guardian was obtained for all participants prior to initiation of study procedures. Randomization (1:1) was stratified by birth weight (< 1000 g and 1000–1500 g) using a randomly permuted block design with random block sizes of 2 and 4 was used to determine the assigned group (intervention versus control). Sequential and sealed numbered envelopes were used. The randomization allocation sequence was generated by team member MC. Participants were enrolled by a designated clinical research coordinator (CRC) and LL. Study team members and participants were blinded to the randomization until after informed consent was obtained. The CRC and LL assigned participants to the group.

The UVA NICU admits an average of 112 very low birth weight infants every year among which 83 are born at 32 weeks’ gestation or less. We anticipated that, in one year of accrual, there would be approximately 120 infants ≤ 32 weeks gestation and/or ≤ 1500 g birthweight. Based on a preliminary data, we expected that 80% of these infants would be eligible for the study and that approximately 80% of eligible infants in this population have abnormal general movement assessments (GMA). This results in approximately 86 patients per year eligible for the study to include abnormal GMAs. Recruitment acceptance rates of previous studies conducted in our NICU range from 30 to 50% for intervention studies and 80–90% for noninvasive studies. An acceptance rate of 80% yields an expected accrual of 70 patients per year. Allowing for 10% dropout, accrual of 33 patients per group would be completed in 1 year.

### Eligibility criteria

Participants were eligible if they were born < 32 weeks and/or < 1500 g, 7 days of age or older; clinically stable (not requiring high frequency mechanical ventilation, vasopressor support, continuous intravenous pain or sedation medication) and if parents (or a suitable surrogate caregiver) had the ability to perform the interventions. Exclusion criteria included known genetic condition impacting neurodevelopment, medically complexity by above definitions persisting by 34 weeks postmentrual age (PMA); non-English-speaking parents; limitations in parental participation with no suitable surrogate caregiver (e.g. incarceration or work/personal related issues). The requirement of having an abnormal GMA was omitted before enrollment began based on another study conducted at by our team that demonstrated that abnormal GMAs are common in preterm infants with poor repertoire being the most prevalent^18^.

Participant demographic and clinical characteristics were abstracted from the electronic medical record. Medical comorbidities included bronchopulmonary dysplasia (defined as oxygen requirement at 36 weeks PMA), sepsis, retinopathy of prematurity (stage 3 or above), necrotizing enterocolitis (unstaged), patent ductus arteriosus requiring pharmacological or surgical treatment as well as neurologic comorbidities including intraventricular hemorrhage (IVH) and white matter injury. IVH was further categorized as low grade IVH (I and II) and high grade IVH (III and IV). High grade IVH was combined with white matter injury due to low prevalence in a relatively small sample for analysis.

### Intervention group

**Figure 1:**
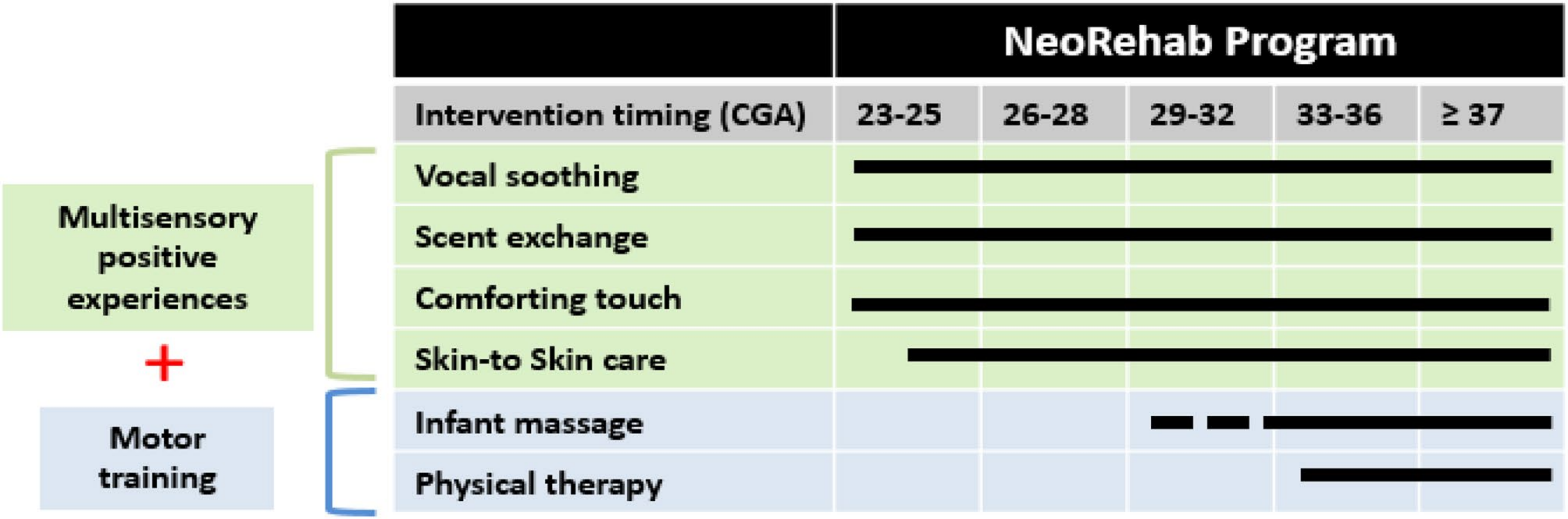
Description of the NeoRehab program. The NeoRehab program centered on 6-interventions (vocal soothing, scent exchange, comforting touch, kangaroo care, infant massage and physical therapy) that parents can provide shortly after birth and that are systematically layered considering the infant’s gestational age and physiologic stability, with increasingly complex motor interventions with advancing postnatal age.

Infants randomized to the NeoRehab program received interventions performed by their parent/caregiver, including: 4 sensory interventions (vocal soothing, scent exchange, comforting touch, skin-to-skin care) as well as 2 motor intensive interventions (infant massage and physical therapy (PT))^19^. All interventions were provided in a GA appropriate fashion and were systematically layered overtime during hospitalization, in addition to usual care. (Fig. 1).

The timing of the 2 motor components was determine by PTs and based on infant GA and clinical stability. Parents met with PT to learn and demonstrate all intervention components. For infant massage parents were instructed to provide a 15 min session twice a day and at least 2 h apart in two phases (moderate pressure tactile and a kinesthetic phases) as previously described^20,21^. The goals of the PT sessions were to promote midline and antigravity play as well as position changes as previously described^20,21^. Parents were instructed to provide two 10 min sessions twice a day, starting at 34 weeks PMA or when deemed clinically appropriate by PT^22^. These three activities incorporate self-discovery of the environment and opportunities to overcome movement difficulties, and build upon the principles of task-specific motor training for infants at high risk for CP^23^.

Parents were provided with oral, written and illustrated information regarding all components of the program. Study team members assisted with demonstrating appropriate technique for applying interventions and used the teach-back method to ensure comprehension. The goal was for the parents to perform all the interventions 5 days per week.

### Usual care group

Parents of infants randomized to usual care were encouraged to touch, hold, and talk to their infants per routine practice; including promotion of skin-to-skin care. Per usual practice, physical and occupational therapists as well as speech language pathologists were consulted on admission for all infants admitted to the NICU. Interventions began when infants were deemed clinically stable and typically included 2–3, 10–30 min sessions per week. Social workers actively engaged with all parents to provide ongoing support and alleviate barriers to visitation. At time of discharge standard care resumed for both groups, which included referral to early intervention services and a NICU follow-up appointment with Developmental Pediatrics at 3 months PMA.

### Outcome measures

The primary outcomes were the acceptability, feasibility and fidelity of the NeoRehab program. Acceptability refers to the view of the intervention and was evaluated using recruitment, refusal, retention, and follow up rates as well as weekly interviews with parental participants. Feasibility refers to the practicality of the intervention of applying the intervention in the NICU setting and was evaluated using direct observations and weekly interviews with parental participants. Fidelity refers to whether or not the interventions were delivered as intended and was assessed using activity logs, direct observations, and weekly interviews.

Self-report activity logs were provided for both groups. Parents in the intervention group were instructed to document what aspects of the program were performed including date as well as type and duration of interventions. Parents in the standard group were instructed to document developmental activities and interactions with their infant. Weekly bedside interviews were conducted with parents in the intervention group to discuss challenges or barriers to performing the NeoRehab interventions until data saturation was achieved. The following questions were included: (1) What is your impression of the program? (2) Do you feel comfortable performing the interventions (3) Are there things that are preventing you from doing the interventions? (4) What are your feelings about how the program/interventions have allowed you to connect with your baby? (5) Do you have any questions about the interventions? Additionally, research team members performed random direct observations of interventions to further evaluate feasibility and fidelity.

### Statistical analysis plan

Frequencies and rates were calculated for categorical variables and means and standard deviations (SD) were to be calculated for continuous variables. Baseline differences were evaluated by group Chi Square was used to evaluate categorical variables and a t-test was used to evaluate continuous variables between groups (Tables 1 and 2). Descriptive statistics were computed for all quantitative variables (demographic characteristics, recruitment, refusal, retention, follow up rates, % completion of self-report log). For those randomized to the intervention group, compliance was measured by calculating, for each person and for each activity, the percentage of weeks the person completed the activity5 or more times. Correlations between feasibility and demographics were evaluated using Spearman rank correlations. Associations between activities and infant or parent characteristics were evaluated using regression models.

**Table 1:** Sample demographic and characteristics based on randomization.

	Entire group (n = 67)	Standard care (n = 33)	Intervention (n = 34)	*p*-value
Gestational age (weeks)	28.38 ± 2.69	28.75 ± 2.68	28.02 ± 2.70	0.272
GA weeks at enrolled	32.16 ± 2.86	31.81 ± 2.77	32.81 ± 2.77	0.334
SGA	17.9%	21.2%	14.7%	0.487
**Gender**				0.353
Male	64.2%	69.7%	58.8%	
Female	35.8%	30.3%	41.2%	
**Race**				0.399
White	74.6%	81.8%	67.6%	
Black	17.9%	12.1%	23.5%	
Hispanic	7.5%	6.1%	8.8%	
**Ethnicity**				0.667
non-Hispanic	92.5%	93.9%	91.2%	
Hispanic	7.5%	6.1%	8.8%	
**Maternal education**				0.298
High school	26.9%	30.3%	23.5%	
GED	11.9%	9.1%	14.7%	
Some college	23.97%	18.2%	29.4%	
College degree	22.4%	18.2%	26.5%	
Post college	13.4%	21.2%	5.9%	
Unknown	1.5%	3%	0%	
**Distance from hospital**				0.580
< 30 miles	16.4%	21.2%	11.8%	
30–60 miles	44.8%	42.4%	47.1%	
> 60 miles	38.8%	36.4%	41.2%	
Received antenatal steroids	91%	100%	82.4%	0.036**
Maternal age	30.26 ± 5.22	30.75 ± 4.71	29.79 ± 4.63	0.455
**Apgar score**				
1 min	5.18 ± 2.52	5.56 ± 2.4	7 ± 2.09	0.237
5 min	6.77 ± 2.11	4.82 ± 2.30	6.55 ± 2.14	0.398
**Indication of delivery**				0.640
Preterm	32.8%	39.4%	26.5%	
Pre-eclampsia	38.8%	33.3%	44.1%	
Fetal	14.9%	15.2%	14.7%	
Abruption	7.5%	9.1%	5.9%	
Other	6%	3%	8.8%	
**Mode of delivery**				0.507
C-section	79.1%	75.8%	82.4%	
Vaginal	20.9%	24.2%	17.6%	
Inborn	82.1%	84.8%	79.4%	0.592
Length of stay	75.58 ± 5357	65.66 ± 36.22	81.20 ± 65.37	0.137
**Disposition**				
Home	98.5%	97%	100%	
Foster	1.5%	3%	0%	
EBM at DC	53.7%	42.4%	64.7%	0.067
**Feeding mode at DC**				0.480
PO	88.1%	93.9%	82.4%	
NG	6%	3%	8.8%	
Gtube	4.5%	3%	5.9%	
GJ tube	1.5%	0%	2.9%	

*DC* discharge, *EBM* expressed breast milk, *GED* general educational development, *GA* gestational age, *SGA* small for gestational age.

*Mean ± standard deviation.

**p-value < 0.05.

**Table 2:** Neurological and medical comorbidities of groups.

	Entire group (n = 67) (%)	Standard care (n = 33) (%)	Intervention (n = 34) (%)	*p*-value
Sepsis	9	6.1	11.8	0.414
Bronchopulmonary dysplasia	26.9	30.3	23.5	0.532
Necrotizing enterocolitis	7.5	9.1	5.9	0.617
Patent ductus arteriosus	10.4	9.1	11.8	0.721
Retinopathy of prematurity	14.9	18.2	11.8	0.461
Intraventricular hemorrhage stage I–II	26.9	27.3	26.5	0.633
Intraventricular hemorrhage grade III–IV or white matter injury	19.4	15.2	23.5	0.386

Direct content analysis was used to analyze the qualitative responses through the use of a priori coding strategy that focused on the overall perceptions, acceptability and feasibility of the NeoRehab Interventions^24^. Specifically the textual data from the interviews and observation notes were analyzed by a primary reviewer (CMD) through (1) immersion in the data (2) followed by line by line analysis and data reduction of the textual data and then a secondary reviewer (JKM) reviewed each except code for agreement. Salient features of the code were aggregated to become inductive themes. Themes were further described through temporal relationship to the amount of time the parents participated in the intervention. Rigor was maintained through documentation of analytic decisions, the use of two coders, and reflexivity practices of the primary coders (who were not directly involved in the intervention).

## Results

### Cohort characteristics

**Figure 2:**
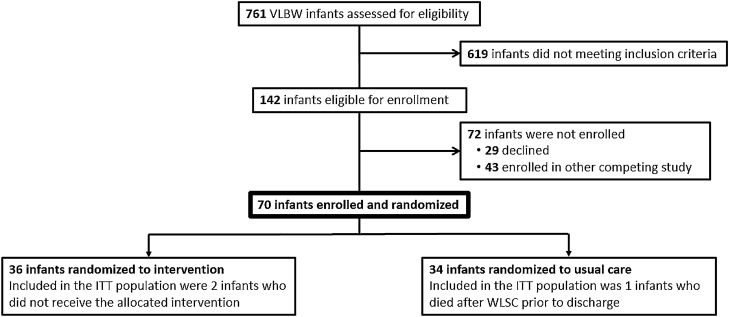
Consort diagram.

Study enrollment occurred from May 2019–October 2020. Of the 761 infants assessed for eligibility during the enrollment period, 691 were excluded. The majority (n = 569) were excluded due to GA and birth weight while 93 were further excluded based on non-English speaking (n = 9); maternal age < 18 (n = 4); post-term when recruitment started (n = 5); social reasons (n = 30); genetic condition (n = 2); withdrawal of life sustaining measures before meeting medical stability criteria (n = 12). Seventy-two infants were appropriate to approach, 29 declined to participate and 43 decided to enroll in a competing study. Seventy participants were consented, 36 were randomized to the intervention group and 34 allocated to usual care (Fig. 2).

The demographic characteristics of the study cohort are outlined in Table 1. The mean GA was 28.3 ± 2.7 weeks and 64.3% of the participants were male. Medical or neurological comorbidities by group are displayed in Table 2. No adverse events were reported during the course of the study.

### Acceptability, feasibility, fidelity of the NeoRehab program

Acceptability outcome metrics included a 71% recruitment rate (73% pre-Covid restrictions to visitation and 65% post-Covid). The retention rate was 98%; two participants withdrew from the study (1 due to personal reasons and 1 due to medical reasons). Primary outcome data were available for all enrolled patients (Fig. 2).

Thirty-six participants were randomized to the intervention group. While we encouraged families to administer the interventions as frequently as possible with the goal of 5 days per week (or 71% of the time), this was determined not to be feasible (Table 3).

**Table 3:** Per patient analyses.

Intervention	≥ 5 days per week	≥ 3 days per week
Vocal soothing	41% (8.7%)	72% (7.9%)
Scent exchange	16% (6.5%)	72% (7.9%)
Comforting touch	41% (8.7%)	75% (7.7%)
Skin to skin	9% (5.1%)	34% (8.4%)
Massage	0% (0%)	6% (4.1%)
Physical therapy	0% (0%)	6% (4.1%)

The % of parents met the pre-established criterion of performing the activities (≥ 5 days per week or ≥ 3 days per week) for at least 71% of the weeks they were on study. 32 participants were randomized to the intervention group with data.

None of the sensory interventions met the 5 days per week pre-established goal. Vocal soothing, scent exchange and comforting touch were performed at least 5 times per week in 41.0 ± 8.7, 16.0 ± 6.6 and 41.0 ± 8.7% of cases, respectively. The fidelity of the program increased to 72.0 ± 7.9, 72.0 ± 7.9 and 75.0 ± 7.7%, respectively when looking at interventions performed 3 days per week. In contrast, no participants were able to provide skin-to-skin care 5 days per week and 34.0 ± 8.4% documented skin-to-skin care at least 3 times per week. Similarly, none of the motor interventions met the goal of 5 days per week and were only performed 3 times per week in 6.0 ± 4.1% of cases. Massage therapy was performed at least 1 time per week in 34% of cases while PT was performed 1 time per week in 22% of cases. Combined massage or PT at least 1 time per week was documented in 34% of patients. When looking at all the NeoRehab interventions combined, 97% of participants documented performing a combination of interventions at least 3 times per week. There was a significant improvement in documentation of the interventions with longer time in the program (*p* = 0.002) and greater birth weight (*p* = 0.019). Maternal variables (age and education) were significantly correlated with documentation of the motor intervention, PT (*p* = 0.04 and *p* = 0.048, respectively), with older and more educated mothers documenting the PT motor intervention more frequently.

The activities logs were also used to provide insight on the fidelity of the interventions performed by the parents. Parents were enrolled for a median of 4 weeks (2–22 weeks) in the study. Thirty-two (88.8%) participants submitted activity log documenting the 6 components of the intervention with their infants, for a total of 190 weeks available for analysis. Documentation was variable and while some parents were diligent about documenting the interventions, others were inconsistent and four participants did not complete the daily activity logs.

### Qualitative evaluation of intervention impact on parents

Several key themes emerged from the qualitative analysis of the acceptability and feasibility of the NeoRehab program (Table 4) and included the following elements: structure of program promotes confidence in care interactions; connection with the child; spillover benefits; clinical considerations for feasibility; and challenges to note.

**Table 4:** Qualitative findings related to acceptability and feasibility of the intervention.

Theme	Qualitative excerpt
Structure of program promotes confidence in care interactions	[It makes me] more comfortable handling the baby when so tiny Helpful to come in to see baby and have the “boxes to check” to know what to do to help the baby Helpful to explain the different techniques used by PT and OT They have taught me a lot of different things to interact with her, sooth her and relax her. I appreciate that very much There was a sense of calm especially for Dad when he was able to have structured direction relating with him The instructions were clear and easy to follow This program makes me feel useful This program seems to give us some ownership in his development
Connection with the child	Program has encouraged to touch, hold, use voice. He is responding to voice and touch When walked in room today he settled so feel like the program is helping her be more connected Scent exchange and skin to skin allows them to be connected Performing the interventions helps me feel close to my baby. He knows I am here for him
Spillover benefits	[The program] is helping me with postpartum depression I was already applying the interventions to my other child, the twin with Downs Syndrome It gave us the feeling that we had some small amount of control over this outcome I feel a necessary part of his team
Clinical considerations for feasibility	We are rarely able to be in the unit for long periods of time. The hardest intervention is kangaroo care because he had a lot of monitors. The massage was difficult to get him prone, especially if the nurse was busy Some of the interventions are not happening often due to her acid reflux and difficulty breathing Baby’s cold temperatures prevent me from undressing him 2 × a day for skin to skin I was not able to perform everything while he was on CPAP
Challenges to note	I keep forgetting to do the scent exchange [Mom] thinks the baby needs to be older to do PT and massage Can’t do the interventions every day because we live more than 2 h away Making sure both parents are trained would be really helpful

Overall, parents considered the delivery of the program as feasible and emphasized that the program structure gave them more confidence in the care interactions with their child. Specifically having components of the intervention that they knew how to do, being educated on the nuances of different techniques, and being provided with clear instructions left the participants with increased confidence in care interactions as well as ownership of their child’s care. One participant reported that “it made me feel useful” and “it makes me more comfortable holding my baby when so tiny.” Further, the program allowed for direct connection with their infant and encouraged touch, holding, voice, and scent exchange in ways that made the participants feel closer to their baby and offered additional tactile modes of connection and soothing. Additionally, participants reported several spillover benefits, or benefits that were not the direct intent of the intervention, but were positive nonetheless. One mother indicated that the program helped her with postpartum depression. Another participant suggested that the knowledge gained allowed them to apply the interventions to their other twin baby at home and even promoted feelings of being a necessary part of the care team. There was also specific feedback that intervention impacted feasibility that were noted due to the complexity of both the care environment and medical complexity of the infant. Several participants noted how specific aspects of the intervention were challenging due to the monitors, technology (i.e., proning a baby with CPAP or mechanical ventilation) or instability of the child (i.e., difficulty breathing, low temperature, etc.). General feasibility concerns like limited parental time in the unit, long distance traveled from home, and both parents needing training were frequently mentioned.

### Parental participation for infants randomized to usual care

Parents were enrolled for a median of 4 weeks (1 to 14 weeks) for a total of 380 cumulative weeks in the study. Twenty seven participants (81.8%) submitted activity logs documenting their interactions with their infant, for a total of 113 weeks available for analysis (30% of total weeks in the study). Documented individual activities included auditory (talking, music, reading), tactile (holding, skin-to-skin, touch, range of motion, massage, diaper change/cares, bathing, feeding and participation in therapy intervention) and olfactory stimuli (scent exchange). Tactile and auditory interventions were the most commonly reported activities (65.6 ± 30.1% and 65.9 ± 28.7%, respectively) followed by scent exchange (45.1 ± 24.4%). With regards to motor specific interventions, 2 participants (7.4%) reported providing infant massage;1–4 times per week for 3–11 weeks and 5 (18.5%) participants documented being present for therapies ranging from 1 to 7 times per week for 1–6 weeks. There was large variability in documentation within the standard care group as completing the daily activity log was not prescribed and left open ended for the parents to report activities using their own judgment.

## Discussion

We found that the NeoRehab program was acceptable with high recruitment (71%) and retention (98%) rates despite visitor restrictions and stress related to the Covid pandemic. Participants reported that the program allowed for direct connection, personal confidence with their infant, and provided them with a purpose during the NICU hospitalization. The goal to perform all interventions 5-days per week was not feasible. Performing a combination of interventions 3-days per week was found to be feasible in 97% of participants of our sample. Interventions performed at least 3 days per week were noted to be feasible for the sensory elements of the program but not the motor elements. Parental prioritization of the motor elements in future studies could be considered once the infant has reached the appropriate GA for intervention. Of note, most parents in our cohort (82.9%) lived more than 30 min away for the NICU with 38.6% residing more than 60 min away, a potential barrier to daily visitation.

The NeoRehab dosing goal is on par with that of other multisensory program where the recommended dosing recommendations vary from daily interventions to 6 h per week. The parental time commitment for education on how and when to provide interventions was comparatively small in our study, with other program requiring upwards of 70 h for education^15,16,25,26^. In contrast to other programs, we strictly focused our analysis on parent-administered interventions excluding interventions provided by health care professionals and/or sensory support team members.

Reliance of the self-report daily activity log to track interventions proved challenging and we likely have an underestimate of the interventions performed for both groups. While parents were provided reminders to complete the documentation of daily activities if randomized to the intervention group, parents often reported that they forgot or lost the paperwork. Parents also reported they would prefer electronic reminders or the ability to complete the documentation electronically versus on paper.

This study sheds more light on the type of interactions parents typically engage in with their infant during NICU hospitalization. Tactile stimuli was the most commonly documented interaction followed by auditory stimulation. Interventions with documented benefit such as skin-to-skin care were underutilized, emphasizing the importance of parents’ education and support to optimize their participation. Parent provided massage and parents being present for therapy intervention were not the norm in the usual care group in our sample. Of note, the documentation was left open-ended for parents, which can allow for variability in reporting. As such, parents may not recognize the simple interactions that they are performing on a daily bases that may in fact be developmentally appropriate and impactful. Further evaluation of the NeoRehab program should consider different thresholds for the different interventions as well as the need to determine the dose needed for motor intervention for infants at highest risk based on standardized assessment (GMA and HINE) and /or brain imaging. Intensive and multimodal parent education sessions may also increase the frequency at which motor interventions are performed. While meeting with PT was required for parents randomized to the intervention group, this proved challenging for parents only able to visit in the evenings after work or on the weekends and they did not have as many opportunities to discuss the motor interventions the PT team. Future studies should include more structured opportunities to interact with the research team and include virtual options, in addition to closely monitoring parental interaction and time spent with PT.

Previous studies indicate that these type of, interventions can be effective in promoting a wide variety of other short-term infant and maternal outcomes^13–17^. We are also currently evaluating short-term developmental outcomes for this cohort which will include the HINE, GMA and TIMP assessments at 3 months corrected GA. Results from this analysis will be key to determine clinical relevance, More refinement of dosing and effectiveness on longer term outcome will be needed to determine if the NeoRehab intervention represents an effective strategy that can widely be implemented.

Limitations of this study include the small sample size and single site design. Additionally, a barrier we identified was a lengthy travel distance for the majority of the sample which likely contributed to the feasibility findings and may not be generalizable to other NICU settings. In addition, patients were enrolled in the study later than intended based on clinical stability criteria. In a future study, we will seek to enroll patients as soon as possible after birth as positive sensory interventions can be systematically applied with adjustments regardless of medical stability. This will allow parents an opportunity to interact with their infant in a safe manner even during critical illness. The sub-optimal self-report of intervention provided by parents was another major limitation of the study, limiting the interpretation of the fidelity data. Significant variability in documentation and potential for missing data and underreporting was noted for participants in both group. In future studies other fidelity measures will be important to consider in addition to parent documentation such as increased presence of study staff at the bedside. A web-based self-report documentation system with routine reminders may also improve adherence. Measurements of fidelity will need to be incorporated into next steps when determining efficacy of the NeoRehab program^27^.

## Conclusion

The UVA NeoRehab program was well received and acceptable to parents of VLBW infants. Programs that place a high demand on parents (5 days per week) are not feasible and goals of intervention at least 3 times per week appear to be feasible in the context of the United States. Parent-provided motor interventions were most challenging to parents and alternative strategies should be considered in future studies.

## Data Availability

The datasets generated during and/or analyzed during the current study are available from the corresponding author on reasonable request.
